# Epigenetic and Epitranscriptomic Regulation of Mastitis in Dairy Cattle: A Review

**DOI:** 10.3390/vetsci13080732

**Published:** 2026-07-24

**Authors:** Shuaishuai Wu, Mohamed Tharwat, Ibrahim F. Halawani, Fuad M. Alzahrani, Khalid J. Alzahrani, Muhammad Zahoor Khan

**Affiliations:** 1 College of Animal Science and Technology, Henan University of Animal Husbandry and Economy, Zhengzhou 450046, China; 2 Department of Clinical Sciences, College of Veterinary Medicine, Qassim University, P.O. Box 6622, Buraidah 51452, Saudi Arabia; atieh@qu.edu.sa; 3 Department of Clinical Laboratory Sciences, College of Applied Medical Sciences, Taif University, P.O. Box 11099, Taif 21944, Saudi Arabia; 4 College of Agriculture and Biology, Liaocheng University, Liaocheng 252000, China

**Keywords:** dairy cattle, bovine mastitis, epigenetic markers, udder health, inflammation, DNA methylation, non-coding RNA

## Abstract

Mastitis remains the most economically damaging disease in the global dairy industry, and conventional genetic selection based on somatic cell score (SCS) has produced only limited gains in resistance. Epigenetic mechanisms—mitotically heritable yet environmentally responsive and largely reversible modifications that regulate gene expression without altering the DNA sequence—are now emerging as a complementary layer of biological information that can sharpen the prediction of disease susceptibility. This review summarizes current evidence on three classes of epigenetic markers associated with bovine mastitis resistance: DNA methylation, non-coding RNAs (with emphasis on microRNAs, long non-coding RNAs, circular RNAs, and small nucleolar RNAs), and histone modifications, alongside the increasingly important epitranscriptomic layer of N6-methyladenosine (m6A) RNA modification. Particular attention is given to differentially methylated regions and discriminant methylation haplotype blocks in immune-related genes, circulating and milk-derived non-coding RNA biomarkers, m6A-mediated regulation of inflammatory transcripts, and histone-mark dynamics in mammary epithelial cells challenged with *Staphylococcus aureus* and *Escherichia coli*. We conclude with current limitations and perspectives on translating these markers into selection tools and therapeutic targets. Across all marker classes, current evidence remains constrained by small cohort sizes, breed-specific study designs, a scarcity of longitudinal and multi-generational data, and limited functional validation, so most candidate markers are still at the discovery stage and require cautious interpretation before deployment.

## Simple Summary

Bovine mastitis remains one of the most economically devastating infectious diseases in dairy production, yet conventional genomic tools capture only a fraction of resistance-trait variance. This review addresses that gap by synthesizing current evidence on epigenetic and epitranscriptomic regulation of mastitis susceptibility—a rapidly advancing field with direct relevance to animal health, welfare, and production efficiency. By critically evaluating DNA methylation markers, non-coding RNAs, m6A modifications, and histone dynamics across multiple pathogens and breeds, this work provides the first integrated framework linking all four regulatory layers to the TLR–NF-κB immune axis in the bovine mammary gland. Readers gain actionable insights into non-invasive biomarker strategies using milk and blood, and a forward-looking research agenda spanning epigenomic selection, pharmacological intervention, and CRISPR-guided epigenome editing. This review is of direct relevance to animal scientists, veterinarians, and breeders striving to reduce antibiotic dependence and improve udder health globally.

## 1. Introduction

Bovine mastitis is an inflammation of the mammary gland, most commonly triggered by intramammary infection with environmental or contagious pathogens [1,2,3]. The disease remains the most prevalent and economically devastating infectious disease in modern dairy production, inflicting estimated annual global losses exceeding USD 35 billion through reduced milk yield, discarded milk, antibiotic treatment costs, premature culling, and compromised animal welfare [4,5,6,7]. The etiological landscape is characterized by two pathogenically distinct microorganisms: *Staphylococcus aureus*, which establishes persistent, frequently subclinical colonization with marked resistance to antimicrobial clearance and is implicated in progressive chronic mammary tissue pathology; and *Escherichia coli*, which precipitates sudden, rapidly progressing acute episodes with potentially fatal clinical outcomes [4,8,9,10]. At the cellular level, host responses to both pathogens are orchestrated through the TLR–NF-κB signaling axis, whose activation induces the expression of pro-inflammatory cytokines, chemokines [11], and antimicrobial peptides in mammary epithelial cells. The magnitude, kinetics, and resolution of this response are pivotal determinants of whether an infection resolves, becomes subclinical, or progresses to chronic tissue destruction [12]. Despite decades of intensive research and the routine use of broad-spectrum antibiotics, mastitis incidence in commercial herds has remained stubbornly high, fueling mounting concerns over antimicrobial resistance and driving an urgent search for durable, genetics-based solutions [13].

Genetic improvement of mastitis resistance has been pursued for more than four decades, yet progress has remained frustratingly incremental relative to the magnitude of the problem. Conventional breeding programs have relied on somatic cell count (SCC) and its log-transformed derivative, the somatic cell score (SCS), as indirect proxies for udder health, but the heritability of these traits rarely exceeds 0.15, fundamentally limiting the rate of genetic gain achievable per generation [14,15]. The advent of high-density SNP genotyping arrays and large-scale genome-wide association studies raised expectations by enabling the identification of dozens of candidate quantitative trait loci linked to udder conformation and clinical mastitis across multiple breeds. Yet despite the scale of these efforts, SNP-based genomic predictions capture only a modest fraction of phenotypic variance in mastitis susceptibility, an observation widely attributed to “missing heritability” that cannot be resolved by larger reference populations or denser marker panels alone. A critical but underappreciated source of this missing heritability lies in the regulatory architecture that converts genotypic potential into infection outcome under real-world conditions of parity, nutrition, stress, and pathogen pressure. Promoter methylation of immune signaling genes, the inducible activity of TLR–NF-κB pathway components, and the post-transcriptional tuning of cytokine networks by microRNAs and long non-coding RNAs are all shaped by factors entirely invisible to SNP arrays, yet they directly determine whether the mammary epithelium mounts a sufficient, appropriately resolved immune response or succumbs to chronic infection [12,16]. Equally, parity-associated and lactation-stage-dependent shifts in mammary methylomes add a further dimension of regulatory plasticity that genotype alone cannot predict. Taken together, these observations compel a fundamental reconceptualization of mastitis resistance: one that moves beyond static DNA-sequence variation and interrogates the dynamic, environmentally responsive epigenomic and epitranscriptomic landscape that actually governs mammary immune competence.

**Figure 1:**
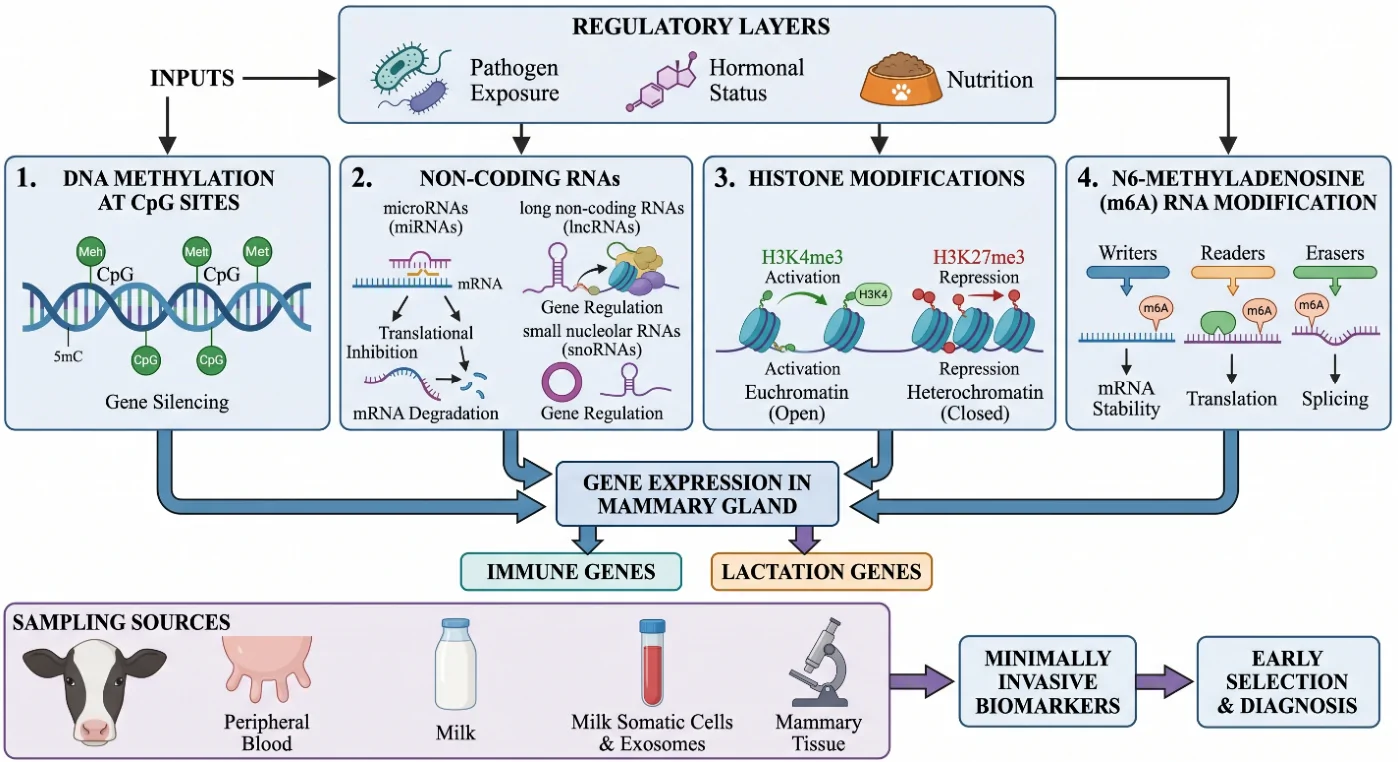
Overview of the epigenetic and epitranscriptomic regulatory layers associated with bovine mastitis resistance. Four classes of marks—DNA methylation at CpG sites, non-coding RNAs (microRNAs, long non-coding RNAs, circular RNAs, and small nucleolar RNAs), histone modifications, and N6-methyladenosine (m6A) RNA modification—respond to pathogen exposure, hormonal status and nutrition to shape the expression of immune and lactation genes in the mammary gland. These markers can be sampled from peripheral blood, milk (somatic cells and exosomes), or mammary tissue, supporting their use as minimally invasive biomarkers for early selection and diagnosis. The four layers differ markedly in the maturity of their supporting evidence. DNA methylation and microRNA markers rest on the broadest experimental and validation base, whereas circular RNA, small nucleolar RNA, m6A, and several histone-mark findings remain largely exploratory and are frequently derived from single studies or computational prediction. The figure therefore depicts a conceptual framework and does not imply equivalent evidentiary support across the four layers.

Epigenetics—the study of mitotically heritable, and in some cases meiotically transmissible, changes in gene expression that occur without alteration of the DNA sequence itself—provides precisely this missing dimension. Evidence accumulated over the past decade has implicated four interacting regulatory layers in the bovine mammary gland response to infection (Figure 1). First, DNA methylation at CpG dinucleotides exerts direct, context-dependent control over the basal and inducible expression of innate immune effectors: hypomethylation of *JAK2* and *STAT5A* promoters in mastitic cows is coupled to their transcriptional activation, whereas hypermethylation of *CD4* suppresses T-helper cell signaling, and these marks show negative correlations with somatic cell count that position them as quantitative epigenetic predictors of disease state [17,18,19]. Genome-wide methylation profiling has extended this principle to the whole-blood methylome, revealing that panels of as few as 50 discriminant CpG positions can reliably distinguish mastitis-resilient from mastitis-susceptible animals within commercial herds [20], and that differentially methylated haplotype blocks in immune-related genes such as *CD48*, *ITGB2*, *SELL*, and TLR pathway components offer breed-independent biomarker signatures [21,22]. Second, non-coding RNAs constitute a post-transcriptional immune regulatory network of remarkable breadth: individual microRNAs such as miR-146b, miR-21, and miR-223 restrain NF-κB-driven inflammation by silencing specific TLR adaptor molecules and cytokine receptors, while ceRNA networks anchored by long non-coding RNAs (including BMNCR and lncRNA-TUB) and circular RNAs act as molecular sponges to fine-tune miRNA availability at key inflammatory nodes. The detection of these species in milk exosomes and peripheral blood creates a minimally invasive diagnostic interface with direct clinical utility [12,23]. Third, the emerging epitranscriptomic layer of N6-methyladenosine (m6A) RNA modification, deposited by *METTL3*/*METTL14* writers and reversed by *FTO/ALKBH5* erasers, regulates the stability and translation of inflammatory transcripts and has now been detected in m6A-modified lncRNAs that constitute a distinct regulatory sublayer within the bovine mastitis transcriptome [24,25,26]. Fourth, covalent histone modifications—particularly HDAC-regulated lysine acetylation and the H3K27me3-targeted demethylase JMJD3—dynamically remodel chromatin accessibility at cytokine and antimicrobial peptide loci during mammary infection, and pharmacological modulation of these marks has already demonstrated proof of concept in preclinical models [27]. The convergence of all four layers on the same TLR–NF-κB–cytokine regulatory axis, combined with their moderate heritability, environmental responsiveness, and accessibility in non-invasive biological fluids, positions the epigenome as a promising, though still largely investigational, source of biomarkers and candidate therapeutic targets for mastitis control. A conceptual distinction must be drawn here, because the term heritable can conflate two separate phenomena: stable transgenerational epigenetic inheritance transmitted across generations of animals, and within-life developmental plasticity, in which marks are reprogrammed in somatic tissues in response to environment, parity, and pathogen exposure. The evidence reviewed below predominantly supports the latter, that is, epigenetic marks behaving as environmentally responsive, mitotically propagated biomarkers of immune state, whereas robust evidence for true transgenerational inheritance of mastitis-associated marks in dairy cattle remains scarce and awaits dedicated multi-generational longitudinal validation.

Against this background, the present review provides a comprehensive and critical synthesis of the epigenetic and epitranscriptomic evidence governing mastitis susceptibility and mammary immune function in dairy cattle, with an explicit focus on translational potential. Section 2 systematically evaluates DNA methylation markers—from locus-specific changes at immune regulatory genes to genome-wide discriminant methylation haplotype blocks—and assesses their suitability for epigenomic selection. Section 3 surveys the full spectrum of non-coding RNA regulators implicated in bovine mastitis, encompassing microRNAs, long non-coding RNAs, circular RNAs, small nucleolar RNAs, and the m6A epitranscriptome, with emphasis on ceRNA network architecture, biomarker accessibility in milk and exosomes, and mechanistic validation. Section 4 integrates the emerging evidence on histone acetylation and demethylase-mediated chromatin remodeling during mammary infection, evaluates current methodological limitations constraining the field, and charts a forward-looking research agenda—spanning multi-omic integration, targeted low-cost assay development, and the incorporation of epigenetic marks into next-generation genomic prediction models—for translating these mechanistic insights into practical tools for mastitis control. The novelty of this review, relative to earlier surveys that have treated non-coding RNAs or DNA methylation in livestock diseases largely in isolation, rests on three features. It integrates all four regulatory layers—DNA methylation, the full non-coding RNA spectrum, the m6A epitranscriptome, and histone modifications—within a single TLR–NF-κB-centered framework rather than reviewing them separately. It explicitly grades the maturity of the underlying evidence, distinguishing mechanistically validated findings from associative or computationally predicted ones. Additionally, it situates these molecular markers within the practical context of dairy herd management, where milking physiology, teat-tissue biomechanics, teat-canal integrity, and automated milking systems govern the environmental and physical pressures to which the mammary epigenome responds [28].

## 2. DNA Methylation Markers

### 2.1. General Mechanism and Relevance to Immune Regulation

DNA methylation in mammals consists of the addition of a methyl group to the 5-carbon of cytosine, primarily at CpG dinucleotides. Promoter hypermethylation is generally associated with transcriptional silencing, whereas gene-body and intronic methylation can have more variable effects on expression. In the context of mastitis, aberrant promoter methylation can alter the basal and inducible expression of innate immune effectors, cytokines, and signaling adaptors, thereby shaping the magnitude and resolution of the inflammatory response in the udder [29,30,31].

Pilot work in dairy cattle has firmly established that DNA methylation is intimately linked to the occurrence and development of acute and clinical mastitis [12,16,18,32]. Promoter hypermethylation of the αs1-casein gene with abrupt shutdown of its expression has been documented in mammary gland tissue of cows with acute *E. coli*-induced mastitis [32], and altered methylomes—predominantly hypermethylation—in bovine mammary epithelial cells exposed to low lipopolysaccharide (LPS) doses (1–10 EU/mL) reshape the expression of milk-production genes such as *ACACA*, *ACSS2*, and *S6K1*, whereas high LPS doses (>10 EU/mL) induce hypomethylation of immune-response genes [33]. DNA methylation rather than genetic mutation has been shown to control *IL-6R* expression in response to mastitogenic pathogens [34], and co-stimulation of bovine mammary epithelial cells with LPS, peptidoglycan, and lipoteichoic acid amplifies hypomethylation and inflammatory transcriptome changes beyond what LPS alone produces, indicating additive effects of multi-PAMP exposure on the mammary methylome [35]. Differentially methylated regions (DMRs) have now been recovered from bovine mammary tissue and peripheral blood challenged with diverse pathogens [21,22,36,37], and genome-wide methylation profiling in the CmCGG context has nominated *IL-6R*, *TNF*, *BTK*, *IL-1R2*, and *TNFSF8* as candidate epigenetic markers of *S. aureus* mastitis [38]. In a mouse model of *S. aureus* mastitis, *NCKAP5* and the transposon *MTD* were among the most prominently differentially methylated loci, further supporting the use of methylation marks as epigenetic indicators of disease [39].

### 2.2. Methylation of JAK–STAT Pathway, CD4, and Related Immune Loci

Among the most thoroughly characterized epigenetic markers in this field are differentially methylated CpG sites in the promoter regions of *JAK2*, *STAT5A*, and *CD4* in Chinese Holstein cattle. Mastitic cows exhibit hypomethylation of the *JAK2* and *STAT5A* promoters, along with increased transcript abundance, whereas the *CD4* promoter is hypermethylated and associated with reduced expression [19,40,41]. Specifically, evaluation of DNA methylation levels in promoter regions revealed that CpG sites within *JAK2* and *STAT5A* were hypomethylated and associated with higher gene expression in cows diagnosed with mastitis compared to healthy controls, whereas the opposite pattern (hypermethylation with lower expression) was observed for *CD4*. DNA methylation was negatively correlated with gene expression across all three genes. Additionally, six, two, and four active transcription factors were identified at CpG sites in the promoters of *JAK2*, *STAT5A*, and *CD4*, respectively. Correlation analysis further demonstrated that *CD4* DNA methylation levels were significantly and positively correlated with somatic cell counts. These three genes regulate prolactin and cytokine signaling and T-helper cell function, and their aberrant methylation has therefore been proposed as a panel of epigenetic predictors of mastitis susceptibility. Overall, findings from this body of work indicate that aberrant DNA methylation at CpG sites within the 1 kb promoter regions of the *JAK2*, *STAT5A*, and *CD4* genes in mastitic cows can serve as potential epigenetic markers for estimating bovine mastitis susceptibility in dairy cattle.

A complementary line of work has extended methylation profiling to peripheral blood—a more accessible matrix than mammary tissue or milk somatic cells—using MeDIP-seq in Xinjiang brown cattle with varying somatic cell counts [41]. Differentially methylated peaks were predominantly located in intergenic regions, followed by introns, exons, and promoters, and 1934 differentially expressed genes were associated with mastitis resistance. Bisulfite-genome sequencing of two priority loci confirmed hypermethylation of *TRAPPC9*—encoding a trafficking-protein-particle complex subunit involved in NF-κB signaling—and concomitant downregulation of *TRAPPC9* in mastitic cows, alongside hypomethylation, but paradoxically reduced expression of *CD4*. Lipopolysaccharide and lipoteichoic acid treatment of MAC-T cells recapitulated the downregulation of *TRAPPC9* in vitro, validating the in vivo observation. The hypomethylated-yet-repressed pattern of *CD4* in the peripheral blood of Xinjiang brown cattle [41] deviates from the canonical inverse promoter-methylation/expression relationship and contrasts with the hypermethylated *CD4* in the peripheral blood of Chinese Holstein cattle reported earlier [18,40]. This breed- and matrix-dependent variability underscores that the link between methylation and transcription is context-dependent, and that the same gene may show opposite methylation signatures across disease stages, cell types, and breeds. Several non-exclusive explanations may reconcile these opposing *CD4* signatures. Biologically, peripheral blood and mammary tissue contain different and shifting proportions of *CD4*-expressing lymphocyte subsets, so a bulk promoter signal reflects cell-type composition as much as per-cell methylation, and acute versus chronic disease stages engage distinct transcriptional programs. Technically, the studies differ in assay chemistry (MeDIP-seq enrichment versus bisulfite or pyrosequencing of individual loci), in the precise CpG positions interrogated, in bisulfite conversion efficiency, and in the sequencing depth available to call methylation, any of which can invert an apparent methylation–expression correlation. Disentangling these contributions will require matched-matrix, cell-type-resolved profiling rather than bulk comparisons across cohorts.

### 2.3. Genome-Wide Methylation Profiling and Resilience Prediction

Enzymatic methyl sequencing (EM-seq) of peripheral blood genomic DNA has enabled genome-wide identification of methylation biomarkers distinguishing resilient from susceptible cows. A landmark study in 12 dairy cows uncovered 196,275 differentially methylated cytosines (DMCs) and 1227 DMRs between resilient and susceptible animals, with key associations with immune-response and morphological-trait genes, including *ENOPH1*, *MYL10*, and *KIR2DL5A* [20]. *ENOPH1* emerged as a particularly important locus, with 19 DMCs identified and methylation marks in both promoter and intronic regions. Quantitative trait loci (QTL) analysis identified body weight as the trait most frequently targeted by DMCs and DMRs. Strikingly, a panel of approximately 50 methylation positions was sufficient to predict resilience in this cohort, providing a preliminary rather than definitive rationale for integrating methylation arrays into early-life selection programs. This result, however, derives from only twelve animals, a sample in which a 50-marker classifier carries a substantial risk of overfitting: when candidate positions vastly outnumber individuals, near-perfect in-sample discrimination can arise by chance and need not generalize. The same caution applies to the discriminant methylation haplotype block panels discussed below, several of which were nominated in cohorts of a few dozen cows. Until these signatures are validated in large, independent, multi-breed populations using held-out test sets, their reported predictive accuracy should be read as an optimistic upper bound rather than expected field performance.

Beyond the Holstein-focused work outlined above, several recent genome-wide methylome studies have substantially enlarged the catalog of mastitis-associated DMRs and discriminant haplotype blocks across breeds, pathogens, and biological matrices. In Vrindavani crossbred cattle, whole-genome methylation sequencing of milk somatic cells from subclinical mastitis-affected animals identified 62,940 DMCs, 7706 DMRs, and 6203 differentially methylated genes (DMGs) compared with healthy controls, with a striking 93.8% of DMCs hypomethylated in the disease state [42]. Methylation loss was not randomly distributed, but followed a structured pattern, preferentially affecting CpG-island shores, shelves, promoters, first introns, and gene bodies, adhering to a well-established inverse relationship between DNA methylation and gene expression. For example, the alarmin gene *S100A8* exhibited 39.5% promoter hypomethylation accompanied by a +2.22 log_2_FC increase in expression, whereas the κ-casein gene *CSN3* showed 24% promoter hypermethylation associated with a −5.12 log_2_FC reduction in expression—a finding further validated by qPCR, which confirmed a −9.6 log_2_FC downregulation. Functional enrichment analysis of hypomethylated, transcriptionally upregulated genes—including *S100A8*, *S100A9*, *CASP1*, and *CCL3*—revealed significant activation of key immune pathways, encompassing B-cell receptor signaling, T-cell receptor signaling, chemokine signaling, Toll-like receptor signaling, Fc receptor-mediated phagocytosis, NETosis, and MHC class II antigen presentation, while hypermethylation-driven silencing systematically suppressed lactational and metabolic programs, as evidenced by the downregulation of *CSN3*, *PCBD1*, *OXCT1*, *PRKAR2B*, and *DNAJB2*. Notably, 51 differentially methylated and expressed genes (DMEGs) were consistently identified across two independent datasets reported by Wang et al., with 235 DMEGs overlapping in one dataset and 128 in the other [43], reinforcing the reproducibility and biological reliability of these methylation–expression associations as potential diagnostic biomarkers [42]. Furthermore, the same study identified key epigenetic regulators—including *GADD45A*, *EXOSC8*, *EYA2*, *PRKAA2*, and *SMARCD3*—as well as the methyltransferase-targeting microRNA *MIR29C* among the DMEGs, suggesting that the inflamed mammary gland not only undergoes widespread epigenetic reprogramming, but also actively remodels the regulatory machinery responsible for maintaining these changes.

A series of *S. aureus*- and *Staphylococcus chromogenes*-focused milk somatic cell methylomes has converged on differentially methylated haplotype blocks (dMHBs) as discriminant signatures. Whole-genome methylome sequencing (WGMS) of 30 Holstein cows (20 subclinical-mastitis, 10 healthy) identified 30,846 dMHBs—76.16% hypermethylated in infected cows—overlapping immune-related genes including *CD48*, *IL10*, *C5aR1*, *FCGR3A*, *IFNG*, *FCER1G*, *FAS*, *TNFAIP8L2*, *CLIC5*, and *EHF*; hypomethylated dMHBs at the *CD48* and *IL10* promoters paralleled gene activation, and integration with transcriptomics linked the dMHB landscape to NF-κB signaling, natural-killer-cell cytotoxicity, and *S. aureus* infection pathways, with 14 dMHBs nominated as candidate discriminant signatures [44]. An expanded WGMS analysis in the same population yielded 3,356,456 DMCs and 153,783 dMHBs, of which 9 dMHBs—including those mapping to *TRAK1*, *MEF2A*, *IL1B*, and *CD81*—discriminated infected from healthy animals; validation in a cohort of 200 cows confirmed significant associations of seven dMHBs with somatic cell score, mammary-gland health, and milk yield [45]. In an *S. chromogenes*-specific design, 53,098 dMHBs were detected (75% hypermethylated), and 16 candidate discriminant dMHBs were anchored on hypermethylated regions of *ZNF691*, *MAP4*, *KCTD6*, *CX3CR1*, *MAML2*, *KCNK5*, *ZC3H7B*, *GATA3*, *LPAR5*, and *PNPLA2* and hypomethylated regions of *DOK3*, *RHOH*, *CD101*, *TSPAN32*, and *IGFLR1*, with inverse methylation–expression correlations observed for *AIF1*, *BATF*, *CCL3*, *CXCL11*, *CXCL17*, *IL12B*, *IL1B*, *IL34*, *IL6*, *IRF6*, *NLRP3*, *NOD2*, *FUT7*, and *CATHL3* [43]. A complementary investigation of *Streptococcus uberis* subclinical mastitis identified global hypomethylation in milk somatic cells (174,342 DMCs, 316 DMRs, and 451 dMHBs), with 1623 methylation–expression-coupled genes enriched in cytokine–cytokine-receptor interaction, chemokine signaling, NF-κB signaling, and antigen processing; key chemokine genes (*CXCL2*, *CXCL8*, *CXCL12*, *CCL3*, *CCL4*, *CCL5*, *CCL20*) were hypomethylated and upregulated, and 26 candidate biomarkers—6 DE genes, 15 CpG-DMCs, and 5 dMHBs—were nominated for resistance breeding [46]. *Streptococcus agalactiae* and *Prototheca* spp., likewise, reshape the milk-somatic-cell methylome, with DMRs in *IL10RB*, *GLT8D2*, *ADAM11*, *IFNAG*, and *IL17REL* that deregulate NF-κB, TLR, RIG-I-like, and energy-metabolism pathways [47]. In contrast, candidate-gene bisulfite sequencing of nine inflammatory loci (*CCL2*, *HCK*, *F11R*, *CD8A*, *PDIA3*, *LGMN*, *HSPA1A*, *IL18*, *NFKBIA*) in Polish Holstein-Friesian cows infected with coagulase-positive or coagulase-negative staphylococci detected only a marginal methylation difference at *HCK* in CoPS-infected animals, suggesting that, at the level of individual candidate promoters, mechanisms other than DNA methylation (notably miRNAs) dominate gene-expression control during chronic mastitis [31].

Methylome-based biomarker discovery has now extended beyond *Bos taurus*. In Mediterranean Italian River buffaloes, nanopore sequencing of peripheral blood DNA revealed 22 mastitis-associated DMCs, with 68% hypomethylated in controls and 32% hypermethylated, and a contrast between intronic enrichment for hypermethylated DMCs and intergenic enrichment for hypomethylated DMCs [48]. These findings represent the first epigenetic overview of mastitis in this buffalo population, although the small sample size positions them as exploratory. A larger MeDIP-seq study of water buffalo peripheral-blood lymphocytes identified 3950 DMRs annotated to 370 DMGs—mostly enriched in promoter regions—with 67 DMGs concomitantly differentially expressed in milk somatic cells, and downregulation of immune genes *CSF2RB*, *C3*, *PZP-like*, and *CPAMD8* in subclinical mastitis; hypomethylation-activated pathways included *Staphylococcus aureus* infection, Th17 cell differentiation, and antigen processing and presentation [49]. Together, these buffalo studies provide the first dedicated epigenomic resources for non-Holstein dairy species, and underscore the broader applicability of methylation biomarkers across the global dairy industry.

Promoter methylation of single, mechanistically tractable loci has also yielded selectable markers. In Xinjiang brown cattle, pyrosequencing identified significantly elevated promoter methylation of the tumor-suppressor *FHIT* in mastitis-affected animals (65.97 ± 19.82% versus 58.00 ± 23.52% in controls, with CpG3, CpG5, CpG8, and CpG15 driving the difference) coupled to reduced *FHIT* expression, alongside slightly reduced methylation of *PIAS1*. Six GWAS-defined resistance loci (*ZRANB3*, *PIAS1*, *ACTR3*, *LPCAT2*, *MGAT5*, *SLC37A2*) anchor this picture, and *FHIT* promoter methylation has been proposed as a target for molecular-marker-assisted selection [50]. A functional proof-of-concept that targeted demethylation can reverse mastitis-associated transcriptional silencing was provided by the application of a pdCas9-C-Tet1-SgRNA 2.0 system at the *AKT1* promoter in *S. aureus*-infected bovine mammary epithelial cells: targeted demethylation restored AKT1 protein expression, reactivated the AKT1/mTOR pathway, rescued milk-protein synthesis and improved cell viability, providing a tractable strategy to alleviate mastitis-associated lactational dysfunction [51]. Extending the epigenetic landscape beyond canonical CpG methylation, a genome-wide screen of *S. aureus*- and *E. coli*-challenged MAC-T cells in vitro and Chinese Holsteins in vivo revealed extensive transposable-element (TE) dysregulation—3046 differentially expressed TEs under *S. aureus* and 22,259 under *E. coli*—operating on immune-related pathways including IL-17 and HIF-1 signaling. Two TE instances from the MER53 (DNA transposon) and MIRc (SINE) families were validated as stably activated and repressed transcriptional markers of *S. aureus* mastitis, expanding the catalogue of epigenetically active markers beyond promoter CpGs [52].

### 2.4. Lactation- and Parity-Associated Methylation Changes

Beyond pathogen exposure, methylation profiles in mammary epithelial cells also shift across lactation stages and parities. Hypomethylation of casein gene promoters (including *CSN1S1*) favors their expression during lactation, illustrating how the same epigenetic machinery that controls milk synthesis can also fine-tune the local immune environment. Multiparous cows exhibit progressive remodeling of mammary methylation patterns that may partly explain the increase in mastitis susceptibility observed with age.

These observations are sharpened by experimental work in murine models. Using reduced-representation bisulfite sequencing (RRBS) on LPS-challenged mammary glands, Ivanova and colleagues [53] showed that inflammation in first lactation (L1-I) and in second lactation with prior inflammation L2-I (L1-I) predominantly produced hypermethylated DMCs and DMRs, whereas inflammation in second lactation without prior inflammation L2-I (L1-I) showed hypomethylation. Key differentially methylated genes included *Slc27a1* and *PPARδ* (PPAR signaling and lipid metabolism), *JDP2* (an NF-κB-mediated transcriptional repressor), and *Egr3* (a prolactin-responsive gene), while *Set7* (encoding an NF-κB-modifying methyltransferase) and *Cxcl1* were upregulated during inflammation, *Wnt4* was downregulated, and *Tlr2* was upregulated in L2-I (L1-I).Strikingly, only one DMR (*Slc27a1*) and two DMCs (*JDP2*) were shared between any two comparisons, and no marks were common to all three, indicating that lactation rank and inflammatory memory profoundly remodel the mammary epigenome, and that mastitis-associated methylation signatures cannot be interpreted without taking parity and prior infection into account [53].

### 2.5. Methodological Heterogeneity and Comparison Across Biological Matrices

The methylation studies surveyed above are not directly comparable, and several of their apparent disagreements reflect methodological rather than biological differences. They span enrichment-based platforms such as MeDIP-seq [41,49], whole-genome and reduced-representation bisulfite sequencing [42,44,45,47,53], enzymatic methyl sequencing [20], targeted bisulfite and pyrosequencing [39,50], and long-read nanopore sequencing [48], each with distinct genomic coverage, resolution, and quantitative behavior, so a region scored as differentially methylated on one platform may be invisible to another. Sample sizes range from a dozen animals to a few hundred [20,45], pathogen models vary from natural subclinical infection to defined experimental challenge with *Staphylococcus aureus*, *Escherichia coli*, *Streptococcus uberis*, or *Staphylococcus chromogenes* [37,38,43,45], and validation ranges from none, through qPCR confirmation [42], to functional perturbation [51]. Because of this heterogeneity, the field has yielded few genuinely replicated marks; the recovery of 51 differentially methylated and expressed genes across two independent datasets stands out as an exception rather than the rule [42,43]. Greater consistency will require harmonized reference methylomes, agreed minimum reporting standards, and pre-registered validation cohorts.

The choice of biological matrix is itself a major source of variability, and the four commonly used sources are not interchangeable. Mammary tissue gives the most direct readout of the infected organ, but requires biopsy and captures a mixture of epithelial, stromal, and immune cells [32,38]. Milk somatic cells are obtained non-invasively and reflect the local inflammatory infiltrate, yet their cellular composition shifts dramatically with somatic cell count, which can confound methylation comparisons between healthy and mastitic quarters [43,45,47]. Peripheral blood is the most accessible matrix and lends itself to early-life screening, but it reports a systemic rather than a mammary signal and may miss locally restricted marks [20,41]. In vitro mammary epithelial cell models such as MAC-T and primary bovine mammary epithelial cells permit controlled mechanistic dissection, but lack the immune and stromal context of the intact gland [33,34,51]. No single matrix has yet demonstrated clear superiority for biomarker discovery, and the same gene can carry divergent methylation across matrices, as the *CD4* example illustrates [18,40,41]. Studies that profile two or more matched matrices from the same animals remain rare, and represent the most informative route toward marks that are simultaneously biologically meaningful and practically samplable [49].

## 3. Non-Coding RNAs and RNA Modifications

Non-coding RNAs (ncRNAs) have emerged as key regulators in bovine mastitis pathogenesis, with mounting evidence from in vivo and in vitro studies highlighting their diagnostic and therapeutic potential, while emerging genome editing technologies and integrated omics platforms offer new avenues for understanding ncRNA-mediated mechanisms and developing improved strategies for mastitis control in dairy cattle [54,55,56,57]. To prevent this breadth from obscuring the most actionable findings, the narrative below prioritizes the limited set of regulators supported by functional validation and convergent cross-study evidence, while the full enumeration of individual differentially expressed species is consolidated in Table 1 and Table 2 rather than in the prose.

### 3.1. MicroRNAs as Post-Transcriptional Regulators of Mammary Inflammation

MicroRNAs (miRNAs) are short (~22-nucleotide) non-coding RNAs that bind predominantly to the 3′ untranslated region of target transcripts, directing translational repression or transcript degradation and thereby fine-tuning the magnitude, kinetics, and resolution of innate and adaptive immune responses. Their abundance in blood, milk, exosomes, mammary epithelial cells, and mammary parenchyma, combined with marked resistance to ribonuclease activity and freeze–thaw cycling, has positioned them as attractive minimally invasive indicators of subclinical infection [58,59]. Across breeds, causative pathogens, and experimental platforms, transcriptomic and small-RNA profiling of healthy versus infected udder tissue has reproducibly identified dozens of differentially expressed miRNAs, of which a compact core set converges on a limited number of innate immune signaling nodes—most prominently the Toll-like receptor (TLR)/NF-κB and JAK-STAT axes.

A coherent mechanistic theme has emerged in which individual miRNAs restrain NF-κB-driven inflammation by silencing discrete adaptors and receptors of the TLR cascade. In lipoteichoic acid (LTA)-stimulated MAC-T cells, miR-146b is induced and directly represses TRAF6, attenuating NF-κB activation and the secretion of TNF-α, IL-1β, and IL-6, whereas its knockdown abolishes this protective effect [60]. An analogous brake is imposed by miR-214 through direct targeting of TRAF1, validated in both bovine mammary epithelial cells (bMECs) and an LPS-induced murine model [61], and by miR-15a, which targets IRAK2 to constitute a candidate miRNA–mRNA predictive pair [62]. miR-125a broadens this paradigm by targeting the IL-6 receptor (IL6R), simultaneously suppressing NF-κB signaling and restoring milk-fat synthesis, thereby coupling inflammatory control to lactational competence via the miR-125a/IL6R/NF-κB axis [63]. Comparable anti-inflammatory activity has been reported for miR-19b, which dampens IL-1β and IL-6 through PI3K-Akt signaling [64], and for the bta-let-7 family—particularly bta-let-7a-5p—which coordinates NF-κB-dependent chemokine and anti-apoptotic gene networks following LPS challenge [65,66]. In streptococcal disease, miR-122 directly targets erythropoietin (EPO) to suppress JAK-STAT signaling, identifying an alternative effector pathway in *Streptococcus agalactiae*-induced mastitis [67]. Counterbalancing these protective regulators, mmu-miR-155 exerts a pro-inflammatory role in *S. aureus* infection and is suppressed by selenium supplementation via inhibition of NF-κB and MAPK phosphorylation [68], while the cell-fate regulators miR-145 (targeting *FSCN1*) [69], miR-223 (targeting *PTPRF*, *DCTN1*, and *DPP9*) [70], and miR-320b (modulating PPAR signaling and ferroptosis) [71] govern the balance between apoptosis, proliferation, and metabolic remodeling that determines tissue repair versus persistent injury.

Beyond direct silencing of inflammatory adaptors, an emerging body of work positions miRNAs at the interface between the methylome and the inflammatory transcriptome, with the regulatory traffic flowing in both directions. Whole-genome methylome profiling of milk somatic cells from cows with subclinical mastitis identified *MIR29C* as a differentially methylated and expressed gene, a finding of mechanistic significance because the miR-29 family directly targets the de novo DNA methyltransferases DNA methyltransferase 3A (*DNMT3A*) and *DNMT3B*, implying that epigenetic dysregulation in the inflamed mammary gland is partly self-sustaining through miRNA-mediated control of the methylation machinery itself [42]. A reciprocal regulatory loop has been described for miR-16b in *S. aureus*-induced mastitis: the pathogen upregulates *DNMT1*, which in turn hypermethylates and silences the miR-16b locus, lifting the repression of its direct target, *YAP1*, and amplifying inflammation. Melatonin reverses this cascade by suppressing *DNMT1*, restoring miR-16b expression, and dampening *YAP1*-driven inflammation in both bMECs and a murine model—a mechanism that nominates the DNMT1/miR-16b/YAP1 axis as a tractable therapeutic node [72]. Together with the *JMJD3*- and *LSD1*-targeted strategies reviewed in Section 4, these findings argue that miRNAs should be viewed not as a parallel layer of regulation, but as integral components of the epigenetic circuitry that determines whether the mammary gland mounts a controlled or a runaway inflammatory response.

Among recurrent regulators, miR-223 is the most consistently implicated across species, biofluids, and disease stages, supporting its candidacy as a robust pan-mastitis biomarker. It is reproducibly upregulated in milk, milk-derived exosomes, and extracellular vesicles during acute and chronic subclinical mastitis [73,74,75], correlates with inflammatory transcripts and California Mastitis Test (CMT) scores [76], and discriminates *Staphylococcus*- and *Streptococcus*-positive milk [77]. It retains diagnostic value in serum of subclinically infected dairy ewes [78], and, when delivered therapeutically via engineered exosomes, alleviates mammary inflammation by repressing RHOB and modulating the TLR4/NF-κB pathway [79]. Computational prioritization of conserved immune regulators independently nominates bta-miR-223 alongside bta-miR-24-3p, bta-miR-149-5p, bta-miR-185, bta-miR-874, and bta-miR-328 as a core hexamer that converges on *TLR4*, *TLR2*, *CXCL8*, and *TNFα*, with bta-miR-223 in particular conserved across 15 species and acting on *CBLB* via the PI3K/AKT/NF-κB axis [80].

Translational interest has centered largely on the use of circulating and secreted miRNAs as non-invasive diagnostics. In milk and milk somatic cells, expression of miR-142-5p, miR-146a, miR-148a, miR-186, miR-383, and the miR-29b family rises in parallel with SCC and CMT score, frequently with high sensitivity and specificity, and in several cases, detects inflammation in CMT-negative or low-SCC samples that escape conventional screening [76,81,82,83,84]. Milk exosomes and extracellular vesicles further enrich this panel: differential cargo of bta-miR-375, bta-miR-378, bta-miR-185, miR-455-3p, miR-503-3p, and the hub regulators bta-miR-2387 and bta-miR-331 have been proposed for early prognosis and disease staging [85,86,87,88], with the small-RNA cargo of milk exosomes in crossbred Vrindavani cattle additionally implicating bta-miR-199a-5p and the novel bta-miR-12030 alongside bta-miR-375, whose downregulation is predicted to derepress the negative immune regulators *CTLA4*, *IHH*, *IRF1*, and *IL7R* [85]. Milk-fat globule and peripheral blood miRNAs (e.g., miR-154c, miR-362-3p, miR-494-3p, miR-1301, and miR-2284r) extend surveillance to compartments amenable to routine sampling [89,90,91]. Importantly, EV miRNA cargo is stable over consecutive days and is specific to the physiological state of the affected quarter, reinforcing its reliability as a longitudinal readout [74].

The miRNA response is also shaped by pathogen identity, indicating scope for etiology-specific diagnostics. *S. aureus* infection consistently dysregulates bta-miR-26a (targeting FGA), miR-664b, miR-23b-3p, miR-331-5p, miR-19b, and miR-2431-3p in mammary tissue [92,93], with LTA-responsive signatures (miR-196a, miR-143) enriched in MAPK signaling [94] and distinct profiles separating coagulase-positive from coagulase-negative staphylococci [95]. *Escherichia coli* challenge elicits a partly overlapping but temporally distinct response (miR-200a, miR-205, miR-122, miR-182), and direct comparison identifies bta-miR-144, bta-miR-451, and bta-miR-7863 as shared markers alongside pathogen-restricted sets [96,97]. Side-by-side challenge of bMECs with *E. coli* and *S. aureus* further nominates miR-149-3p and miR-1777b as cross-pathogen regulators acting through pathogen-specific lncRNA partners (LOC100140121 and LOC104971359 for *E. coli*; LOC112442703 and LOC104971369 for *S. aureus*), with predicted targets including *MAPK3*, *MAPK14*, *PIK3R2*, *RELA*, *NOTCH2*, and *JAK3*—collectively converging on cell-cycle arrest, ROS production, apoptosis, and IL-1β/IL-6/TNF-α induction [98]. In macrophages, *S. agalactiae* strains differentially drive miR-146b, miR-155, and macrophage-polarization miRNAs, influencing the M1/M2 balance and bacterial clearance [99].

Beyond single miRNA–target relationships, systems-level reconstructions have clarified how non-coding RNAs operate within integrated regulatory circuits. Competing endogenous RNA (ceRNA) and miRNA–lncRNA–mRNA networks reveal that anti-inflammatory miRNAs such as bta-miR-30a-5p, bta-miR-125a, and bta-miR-193b target core cytokine and TLR4–NFKB1–STAT3 hubs, that lncRNAs including CDC42SE1 act as hub regulators of inflammatory features, and that dysregulation of bta-miR-149 and bta-miR-615 sustains pathogen colonization [100,101]. Comparable ceRNA architectures have been described in Xinjiang brown cattle, where bta-miR-2415-3p, bta-miR-3431, bta-miR-2904, and several novel miRNAs (novel_171, novel_348, novel_575) participate in lncRNA- and circRNA-anchored networks that converge on PI3K-Akt signaling, focal adhesion, ECM-receptor interaction, chemokine signaling, and cell-adhesion molecule pathways [102]. A discrete ceRNA loop has also been functionally validated in *S. aureus*-infected bMECs, in which the upregulated lncRNA CMR sponges miR-877 to relieve repression of FOXM1; knockdown of CMR suppresses proliferation, induces apoptosis, and curbs inflammatory output, effects that are reversed by either miR-877 overexpression or *FOXM1* inhibition, establishing CMR/miR-877/FOXM1 as a self-protective autoregulatory axis in mammary epithelium [103]. A genetic dimension is added by sequence variants within miRNA loci: a seed-region SNP in bta-miR-2899 alters its repression of the immune regulator SPI1 and associates with somatic cell score, while integrative GWAS/QTL analysis links miR-223 target genes to mastitis-resistance loci—together nominating functional miR-SNPs as selectable markers for resistance breeding [70,104]. Predisposing stressors are likewise reflected in the miRNome, with heat stress modulating bta-miR-21-5p, bta-miR-146b, and bta-miR-145 through Wnt, TGF-β, MAPK, and JAK-STAT pathways relevant to inflammatory susceptibility [105].

While the miRNA literature is now extensive, an adjacent class of small non-coding RNAs—PIWI-interacting RNAs (piRNAs), 26–31 nucleotides in length—is beginning to attract attention as a potentially overlooked regulatory layer in mammary inflammation. Although research on livestock piRNAs remains in its infancy, evidence from model organisms and human breast pathology indicates that piRNAs modulate inflammatory progression and stem-cell proliferation, raising the prospect that they participate in mammary gland development and in the onset or resolution of mastitis. Their regulatory networks, therefore, represent a plausible but largely unexplored target for novel diagnostic and therapeutic strategies in dairy cattle [106].

Collectively, these data establish miRNAs as both mechanistic governors and accessible reporters of mammary inflammation. The convergence of independent studies on a core set of regulators—chief among them miR-223, miR-146a/b, and the let-7 family acting through TLR/NF-κB signaling—lends biological coherence to an otherwise heterogeneous literature and provides validated targets for diagnostic panels and nucleic acid therapeutics. The integration of miRNAs with the methylation machinery (via miR-29C–*DNMT3A/3B* and the *DNMT1*/miR-16b/YAP1 axis), with lncRNA- and circRNA-anchored ceRNA networks (CMR/miR-877/FOXM1; the Xinjiang brown cattle networks), and with the emerging piRNA layer further argues that future biomarker panels and breeding strategies will need to capture this multi-tiered non-coding RNA landscape rather than individual miRNAs in isolation. Nonetheless, current evidence is tempered by modest cohort sizes, variable diagnostic performance (ROC AUCs frequently in the 0.70–0.74 range for individual serum miRNAs), and inconsistent normalization across platforms, underscoring the need for large, standardized, multi-breed validation before clinical or breeding deployment. In practical terms, only miR-223, miR-146a/b, and the let-7 family currently satisfy the dual requirement of mechanistic validation and reproducible cross-cohort detection demanded of a deployable biomarker, and the remaining species catalogued in Table 1 are best regarded as supporting candidates pending independent replication.

**Table 1:** Differentially expressed and functionally validated miRNAs in mastitis animal mammary gland tissue.

miRNA(s)	Regulation in Mastitis	Model/Sample	Pathogen/Stimulus	Validated Target(s)/Pathway	Principal Finding and Proposed Utility	Reference(s)
bta-miR-30a-5p, -125a, -193b, -149, -615, etc.	Mixed	Network/tissue	Mixed incl. *Str. uberis*	TLR4-NFKB1-STAT3, TNF, IL10; lncRNA CDC42SE1	Integrated regulatory networks; diagnostic/therapeutic targets	[100,101]
bta-let-7a-5p, miR-30a-5p, miR-125b, miR-100	Differential	bMECs	LPS	CXCL1/3/6, IL8, BCL2A1, BIRC3; NFKBIA	Co-ordinate inflammation and anti-apoptosis	[65,66]
MIR29C	Differentially methylated and expressed	Milk somatic cells (SCM)	Subclinical mastitis	DNMT3A/DNMT3B	miRNA-mediated control of de novo methylation machinery; self-sustaining epigenetic dysregulation	[42]
miR-146b	↑	MAC-T	LTA	TRAF6 mediated NF-κB activation	↓TNF-α/IL-1β/IL-6; therapeutic anti-inflammatory target	[60]
miR-21, miR-223	↑	Serum (Lacaune ewes)	*S. aureus* (SCM)	—	Non-invasive SCM biomarkers; miR-223 AUC 0.737	[78]
miR-125a	↓	bMECs, mammary tissue, mouse	LPS	IL6R mediated NF-κB activation	miR-125a/IL6R/NF-κB axis links inflammation and milk-fat synthesis	[63]
miR-223	↑	MAC-T	*S. aureus*	PTPRF, DCTN1, DPP9, CDC25B	Represses apoptosis/necrosis; target genes enriched in SCC/mastitis QTLs—breeding markers	[70]
miR-320b	↑	bMECs	LPS	PPARγ, FABP4, LPL; ↓COX-2, IL-12A, iNOS, MAPK1/14	Suppresses inflammation and modulates lipid metabolism (ferroptosis, PPAR)	[71]
miR-148a, miR-186	↑	Buffalo milk	SCM	—	Positively correlate with SCC; SCM biomarkers in buffalo	[81]
miR-223-3p, miR-26-5p	↑/↓	Milk, immune cells	Mixed (staging)	—	Combined with SCC, stage acute vs. chronic mastitis	[75]
miR-214	↓	bMECs, mouse	LPS	TRAF1 mediated NF-κB activation	Anti-inflammatory; alleviates pathological damage in vivo	[61]
bta-miR-199a-5p, bta-miR-12030 (with bta-miR-375)	Differential	Milk exosomes (Vrindavani crossbred)	SCM	CTLA4, IHH, IRF1, IL7R (predicted)	Non-invasive exosomal panel for early SCM diagnosis	[85]
bta-miR-2415-3p, bta-miR-3431, bta-miR-2904, novel_171/348/575	Differential (57 DE-miRNAs total)	Mammary tissue (Xinjiang brown cattle)	Clinical mastitis	PI3K-Akt, focal adhesion, ECM-receptor, chemokine signaling	ceRNA hubs (lncRNA-/circRNA-anchored) for mastitis resistance	[44]
miR-877	Suppressed by lncRNA CMR sponging	bMECs	*S. aureus*	FOXM1	CMR/miR-877/FOXM1 autoprotective axis; therapeutic target	[103]
miR-16b	↓ (silenced by DNMT1 hypermethylation); restored by melatonin	bMECs, mouse	*S. aureus*	YAP1; DNMT1/miR-16b/YAP1 axis	Melatonin reverses pathogen-driven methylation silencing; therapeutic node	[72]
bta-miR-223, bta-miR-24-3p, bta-miR-149-5p, bta-miR-185, bta-miR-874, bta-miR-328	Computationally prioritized	In silico/network	Mammary gland inflammation	TLR4, TLR2, CXCL8, TNFα, CBLB; PI3K/AKT/NF-κB	Core hexamer of immune-regulatory miRNAs; ceRNA partners include XR_003033296.1 and XR_234647.4	[80]
miR-149-3p, miR-1777b	Differential	bMECs	*E. coli* and *S. aureus*	MAPK3, MAPK14, PIK3R2, RELA, NOTCH2, JAK3	Cross-pathogen lncRNA-partnered regulators of TLR4/NF-κB output	[98]
piRNAs (class)	Emerging field	Livestock/human/model organisms	Multiple	Inflammation, stem-cell proliferation	Underexplored small ncRNA layer in mammary health	[106]
bta-miR-144, -451, -7863	Differential	Mammary gland	*E. coli* vs. *S. aureus*	TLR, MAPK, TGF-β, chemokine	Shared cross-pathogen diagnostic biomarkers	[97]
bta-miR-21-5p, -99a-5p, -146b, -145, -133a, -29c	Mostly ↑	Mammary tissue	Heat stress	Wnt, TGF-β, MAPK, Notch, JAK-STAT	Heat-stress regulators; mastitis predisposition	[105]
bta-miR-146b, -221, -222, -155, -125a/b	Up/down	Monocyte-derived macrophages	*S. agalactiae* (ST103/ST12)	Macrophage polarization, TLR2	Strain-specific M1/M2 regulation; SCM markers	[99]

Abbreviations: bMECs, bovine mammary epithelial cells; CM, clinical mastitis; CMT, California Mastitis Test; CoPS/CoNS, coagulase-positive/negative staphylococci; EV, extracellular vesicle; LPS, lipopolysaccharide; LTA, lipoteichoic acid; MFG, milk-fat globule; QTL, quantitative trait locus; SCC, somatic cell count; SCM, subclinical mastitis; SCS, somatic cell score. ↓ represents suppression/down-regulation while ↑ shows up-regulation/overexpression.

### 3.2. Long Non-Coding RNAs and RNA m6A Modification

Long non-coding RNAs (lncRNAs) have emerged as central regulators of inflammatory homeostasis in the bovine mammary gland, and a growing body of work positions them—together with epitranscriptomic N6-methyladenosine (m6A) modification—as integral components of mastitis biology [23]. Transcriptomic surveys consistently identify hundreds to thousands of differentially expressed lncRNAs in pathogen-challenged tissues or cells, indicating that lncRNA dysregulation is a pervasive, rather than incidental, feature of intramammary infection. Across the literature, mechanistic studies converge on four broad themes: (i) competing endogenous RNA (ceRNA) sponging of miRNAs, (ii) modulation of canonical NF-κB and MAPK inflammatory cascades, (iii) regulation of epithelial barrier integrity and host–pathogen adhesion, and (iv) cross-talk with m6A-based RNA modification. These themes are not mutually exclusive; many individual lncRNAs operate at the intersection of two or more, and the same lncRNA can assume protective or pathogenic roles depending on the stimulus, dose, and cellular context.

ceRNA networks as a recurring regulatory motif:. Among the most reproducible findings in bovine mastitis is the operation of lncRNAs as molecular sponges for miRNAs, derepressing downstream pro- or anti-inflammatory targets. BMNCR exemplifies this paradigm: in *S. aureus*-infected mammary tissue and bMECs, BMNCR is strongly upregulated and engages bta-miR-145, with two complementary downstream effectors reported—ANO6 [17] and CBFB [107]—both shaping proliferation, apoptosis, and pro-inflammatory cytokine output (IL-2, IL-6, IL-8, IL-12). A similar ceRNA architecture underpins the lncCRHR1/miR-302d/FGF19 axis in disease-resilient Xinjiang Brown cattle, where cytoplasmic lncCRHR1 promotes MAC-T proliferation and cytokine release while suppressing apoptosis [108], and the CMR/miR-877/FOXM1 axis, where CMR knockdown attenuates the inflammatory response of *S. aureus*-challenged bMECs [103]. The CA12-AS1/miR-133a axis adds a further layer, simultaneously controlling cytokine secretion, NF-κB activity, tight-junction gene expression, and apoptosis [109]. Genome-wide ceRNA network reconstructions in Xinjiang Brown cattle [44], and bovine monocytes challenged with *Streptococcus uberis* [101] confirm that such axes are not isolated curiosities, but pervade the mastitic transcriptome, with multiple lncRNAs converging on a relatively small set of inflammation-relevant miRNAs (notably miR-145, miR-149, miR-133a, miR-877, and miR-302d).

NF-κB and MAPK signaling as a downstream choke-point: Functional perturbation studies consistently show that pro-inflammatory lncRNAs amplify mastitis severity by interacting with NF-κB and/or MAPK signaling pathways. HULIB binds directly to *PP2AB*, enhancing NF-κB activity via upregulation of TLR4 and NF-κB1, and driving expression of IL-6, IL-8, IL-1β, and apoptosis-related genes (*BAX*, *CASP9*, *CASP3*), while suppressing *PCNA*, *Cyclin D1*, and *CDK4* [110]. lnc-ANRIL likewise potentiates NF-κB-dependent cytokine output in LPS-treated MAC-T cells [111], and TCONS_00058979—the first lncRNA shown to associate biochemically with components of the NF-κB and MAPK pathways—exacerbates LPS-induced bMECs damage in a dose-dependent manner [112]. A converse pattern is observed for exosomal lnc-AFTR, which is downregulated in *S. aureus*-induced mastitis and acts as a translational repressor of *FAS* mRNA, thereby suppressing Caspase-8/3 and JNK activation by inhibiting TNF and MAPK signaling [113]. Time-resolved profiling of LPS-stimulated MAC-T cells further distinguishes pathogenic from protective species within a single dataset, with TCONS_00139850 amplifying inflammation, whereas TCONS_00039271 appears to restrain mastitis development [114].

Barrier integrity and host–pathogen adhesion: A complementary group of lncRNAs links transcriptional regulation to physical defense of the epithelium. lncRNA-TUB, predicted to target *TUBA1C*, is induced under pro-inflammatory stimulation and shapes the morphology, proliferation, migration, and β-casein secretion of mammary epithelial cells, while also mediating *E. coli*-driven cytokine release and *S. aureus* adhesion [115]. *LRRC75A-AS1*, the antisense partner of *LRRC75A*, is downregulated in *E. coli*-challenged mammary tissue; its CRISPR-mediated knockout reinforces tight-junction proteins (Claudin-1, Occludin, ZO-1), reduces monolayer permeability, and inhibits *S. aureus* invasion [116]. H19 presents a particularly informative dual phenotype: under baseline conditions, it sustains proliferation, β-casein production, and tight-junction integrity, thereby restricting *S. aureus* adhesion, but under LPS challenge, it amplifies NF-κB-driven cytokine release [117]. These observations argue against a strictly binary pro- versus anti-inflammatory categorization of lncRNAs and suggest that physiological context—pathogen identity, dose, and developmental state—dictates their net effect.

Genome-scale discovery and resistance-trait mapping: High-throughput RNA-sequencing has progressively expanded the catalogue of mastitis-associated lncRNAs from a handful of mechanistically characterized loci to several thousand differentially expressed transcripts: 94 in milk somatic cells of healthy versus mastitic cows [118], 231 in LPS-treated bMECs [119], 270 in *S. aureus*-infected Holstein tissue [120], 1757 in clinically mastitic Xinjiang Brown cattle [102], and 2597 in bMECs challenged with *E. coli* or *S. aureus* [98]. Many of these transcripts—LOC107133214, LOC104974443, LOC101906793, LOC112449280, and related loci—interact with NOD-like, TNF, and MAPK signaling pathways by targeting *IL-6*, *NFKB1*, *TNFAIP3*, *CCL2*, and *CXCL8* [119]. Crucially, integration with QTL and GWAS data has begun to identify lncRNAs of selective interest for breeding: *PRANCR* and *TNK2-AS1* emerge as stable markers across in vivo and in vitro *S. aureus* models and respond favorably to folic acid supplementation [121]; MSTRG.11108.1/ICAM1 is linked to somatic cell count under folic acid challenge [122]; and 37 of 70 differentially expressed lncRNAs in peripheral blood map to mastitis QTLs—including MSTRG25101.2, MSTRG.56327.1, and MSTRG.18968.1, which target *TLR4*, *NOD2*, *CXCL8*, and *OAS2* [123]. Computational and cross-species mining further nominate lncRNAs such as NONBTAT027932.1 and XR_003029725.1 as hubs in lncRNA–miRNA–TF–mRNA networks regulating *TLR2/4*, *CXCL8*, and *TNFα* [80], while WGCNA of *Streptococcus uberis*-challenged bovine monocytes identifies five core lncRNAs (e.g., ENSBTAG00000048401) co-expressed with key miRNAs and transcription factors (*SOX10*, *MYCL*, *ETV4*, *MAFB*) that govern leukocyte immunity, TLR2 signaling, and p38 MAPK activation [101].

Cross-talk with m6A modification: Layered atop lncRNA-mediated regulation, m6A—the most abundant internal modification of mRNA—has been profiled across mastitis models and identified as a non-coding RNA-adjacent epitranscriptomic axis. In MAC-T cells exposed to heat-inactivated *S. aureus*, 133 hypermethylated and 711 hypomethylated genes are enriched in oxidative stress, lipid metabolism, and inflammatory pathways, with 62 displaying concordant changes in mRNA abundance [98]. Mechanistically, the m6A reader *YTHDF2* is downregulated during *S. aureus* infection, destabilizing m6A-modified *IER3* mRNA and driving ROS accumulation, mitochondrial dysfunction, and apoptosis; restoration of YTHDF2 rescues these phenotypes and is therefore proposed as a candidate marker for resistance breeding [124]. Critically, m6A also operates on lncRNAs themselves: MeRIP-seq of *S. aureus*-injured bMECs identified 140 differential m6A peaks corresponding to 130 lncRNAs enriched in WNT signaling, amino acid metabolism, and metalloproteinase activity, providing the first evidence that m6A-modified lncRNAs constitute a distinct regulatory layer in mastitis [25]. Together, these observations indicate that lncRNA function in the mastitic mammary gland cannot be fully understood without considering the epitranscriptomic context in which lncRNAs are written, read, and turned over.

Synthesis: The lncRNA-centered literature delineates a multi-tier regulatory architecture in which a relatively small number of mechanistically deep loci (BMNCR, lncRNA-TUB, LRRC75A-AS1, H19, HULIB, CMR, lnc-AFTR, CA12-AS1) operate within a much broader landscape of transcriptionally responsive species [17,103,107,109,110,115,116,117]. ceRNA logic explains how individual lncRNAs leverage shared miRNA pools to coordinate inflammation, proliferation, apoptosis, and barrier maintenance; convergence on NF-κB and MAPK signaling rationalizes the consistent functional read-outs across pathogens and stimuli; and emerging m6A data [25,98,124] place lncRNAs within an epitranscriptomic framework. The reproducible recovery of mastitis-associated lncRNAs in QTL- and GWAS-anchored studies [121,122,123] makes them attractive both as diagnostic biomarkers (particularly in peripheral blood and milk somatic cells) and as targets for genomically informed breeding of mastitis-resistant dairy cattle. A summary of the key lncRNAs and m6A regulators discussed above is provided in Table 2.

**Table 2:** Long non-coding RNAs and m6A regulators implicated in bovine mastitis: model systems, mechanistic axes, and functional roles.

lncRNA/RNA Modification Factor	Model/Pathogen/Stimulus	Mechanism/Target Axis	Expression in Mastitis	Functional Role	Reference
lncRNA-TUB	bMECs; pro-inflammatory stimulation; *E. coli*; *S. aureus*	Predicted to target *TUBA1C*; CRISPR/Cas9 knockout	Upregulated	Regulates proliferation, morphology, migration, and β-casein secretion; mediates *E. coli*-induced cytokine release and *S. aureus* adhesion	[115]
lncCRHR1	MAC-T cells; Xinjiang Brown cattle	lncCRHR1/miR-302d/FGF19 ceRNA (cytoplasmic)	Upregulated	Promotes proliferation and inflammatory cytokine release; suppresses apoptosis	[108]
BMNCR	Holstein mammary tissue and bMECs; *S. aureus*	Sponges bta-miR-145 suppresses *ANO6* expression	Upregulated	Knockdown impairs proliferation, promotes apoptosis, alters IL-2/IL-6/IL-8/IL-12	[17]
BMNCR	*S. aureus* mastitis	Sponges bta-miR-145 activates CBFB	Upregulated	Pro-inflammatory; increases apoptosis, inhibits proliferation; knockdown enhances IL-1α/2/6/8/12	[107]
TCONS_00058979	bMECs; LPS	Associated with components of NF-κB and MAPK pathways activation	Upregulated	Pro-inflammatory; exacerbates IL-1β/IL-6/IL-8 and apoptosis	[112]
HULIB	LPS-induced bMECs (cytoplasmic)	Binds *PP2AB* and upregulates TLR4/NF-κB1	Upregulated	Pro-inflammatory; ↑IL-6/IL-8/IL-1β and BAX/CASP9/CASP3; ↓PCNA/Cyclin D1/CDK4	[110]
94 DE lncRNAs (e.g., ENSBTAG00000070418_2, ENSBTAG00000082333, lincRNA_64.1, lincRNA_2411.6)	Healthy vs. mastitic milk somatic cells	Target *PIK3R4, CSN3, COBLL1, TNFRSF1A*	Mixed	Inflammatory and immune-response pathways of the mammary gland	[118]
CA12-AS1	LPS-induced bMECs	Negatively correlated with and directly targets miR-133a	Upregulated	Pro-inflammatory; ↑NF-κB, BAX, caspase-3/9; ↓Claudin-1/Occludin/ZO-1, CDK2/4, PCNA	[109]
231 DE lncRNAs (LOC107133214, LOC104974443, LOC101906793, LOC112449280, LOC112448073, LOC112444516)	LPS-induced bMECs	Regulate *IL-6, NFKB1, TNFAIP3, CCL2, CXCL8, RELB*	Differentially expressed	NOD-like/TNF/MAPK signaling; inflammation, pyroptosis, apoptosis	[119]
MSTRG.498 (and MSTRG.57.1, MSTRG.41.1, MSTRG.124.1)	Mammary tissue; *S. aureus*-infected Holstein cows	MSTRG.498 targets *SMC4*; ErbB/hydrolase activity	Differentially expressed	Mastitis pathogenesis via *SMC4*; embedded in lncRNA–miRNA–mRNA network	[120]
MSTRG.11108.1	Subclinical mastitic cows ± folic acid	Targets *ICAM1, CCL3, CCL4*; co-localized with SCC/mastitis QTL	Modulated by folic acid	Folic-acid-mediated immune enhancement	[122]
lnc-ANRIL	MAC-T cells; LPS	Regulates inflammatory cytokines via NF-κB	Pro-inflammatory (knockdown protective)	Knockdown promotes proliferation, reduces apoptosis and immune activation	[111]
lnc-AFTR (exosomal)	Exosomes/MAC-T/mastitis tissue; *S. aureus*	Binds FAS mRNA and blocks translation; inhibits Caspase-8/3 and JNK	Downregulated	Protective: ↓apoptosis, ↑proliferation; suppresses TNF and MAPK	[113]
TCONS_00039271 (↓)/TCONS_00139850 (↑)	MAC-T cells; LPS time-course (0/6/12 h)	Notch, NF-κB and PI3K-Akt	Opposing regulation	TCONS_00139850 promotes inflammation; TCONS_00039271 may suppress mastitis	[114]
PRANCR, TNK2-AS1	Holstein tissue + MAC-T; *S. aureus* ± folic acid	PRANCR regulates *SELPLG* and *ITGB2*; surrounding SNPs link to immune traits	Stable differential expression	Conserved markers; folic acid restores expression	[121]
LRRC75A-AS1	MAC-T and mammary tissue; *E. coli*; CRISPR KO	Cis-regulates LRRC75A and tight-junction proteins; modulates NF-κB	Downregulated	Protective: KO enhances Claudin-1/Occludin/ZO-1, reduces permeability and *S. aureus* invasion	[116]
H19	MAC-T cells; baseline vs. LPS and *S. aureus*	Tight-junction maintenance and NF-κB activation (dual)	Upregulated under LPS	Sustains proliferation, β-casein, and barrier; amplifies TNF-α/IL-6/CXCL2/CCL5 under LPS	[117]
CMR	*S. aureus*-induced bMEC mastitis model	Sponges miR-877 and upregulates FOXM1 (ceRNA)	Upregulated	Pro-inflammatory; knockdown inhibits proliferation, induces apoptosis, reduces cytokines	[103]
MSTRG25101.2, MSTRG.56327.1, MSTRG.18968.1 (70 DELs)	Peripheral blood; subclinical mastitis	Predicted cis/trans regulation of *TLR4*, *NOD2*, *CXCL8* and *OAS2*	Differentially expressed	37 of 70 DELs co-localise with mastitis QTL (SCS, SCC)	[123]
8 lncRNAs (e.g., NONBTAT027932.1, XR_003029725.1)	Computational/integrative bovine mastitis analysis	ceRNA with bta-miR-223, miR-149-5p, miR-24-3p; TLR4/TLR2, CXCL8, TNFα	Predicted regulators	Hub nodes in lncRNA–miRNA–TF network of innate immunity	[80]
2597 lncRNAs (LOC100140121, LOC104971359, LOC112442703, LOC104971369…)	bMECs; *E. coli* and *S. aureus* injury	Bind miR-149-3p and miR-1777b → and regulates *MAPK3/14*, *PIK3R2*, *RELA*, *NOTCH2*, *JAK3*	Differentially expressed	ceRNA control of TLR4/NF-κB, cell cycle, ROS, apoptosis, cytokines	[98]
1757 DE lncRNAs (TCONS_00211035, TCONS_00114426, TCONS_00612301, TCONS_00047055, TCONS_00062142)	Xinjiang Brown cattle (healthy vs. clinical mastitis)	ceRNA (lncRNA–miRNA–mRNA); PI3K-Akt, focal adhesion, chemokine signaling	Differentially expressed	Hub genes *CSF1R, RHO, RCVRN, CAV3, GATA4*	[44]
5 lncRNAs (ENSBTAG00000048401, …049095, …05046, …051337, …051777)	Bovine monocytes; *Streptococcus uberis* (WGCNA)	Co-expression with miR-149/miR-615/miR-133a and TFs (*SOX10, MYCL, MAFB, ETV4*)	Differentially expressed	Leukocyte immunity, TLR2 signaling, B/T-cell activation, p38 MAPK	[101]
m6A landscape (133 hyper-, 711 hypo-methylated genes; 62 with concordant mRNA changes)	MAC-T cells; heat-inactivated *S. aureus*	Transcriptome-wide m6A profiling	Hyper- and hypomethylation	Oxidative-stress, lipid-metabolism, inflammatory pathways	[98]
YTHDF2/IER3 (m6A reader axis)	bMECs; *S. aureus*	YTHDF2-dependent stability of m6A-modified IER3 mRNA	YTHDF2 downregulated	Loss destabilizes IER3 → ↑ROS, mitochondrial dysfunction, apoptosis	[124]
m6A-modified lncRNAs (140 peaks, 130 lncRNAs)	bMECs; heat-inactivated *S. aureus*; MeRIP-seq	WNT signaling; amino-acid metabolism; metalloproteinase activity	Differentially methylated	First evidence that m6A-modified lncRNAs constitute a distinct regulatory layer	[25]

Note: ↓ represents suppression/down-regulation while ↑ shows up-regulation/overexpression.

### 3.3. Circular RNAs, Small Nucleolar RNAs, and Additional Epitranscriptomic Regulators

Beyond miRNAs and lncRNAs, recent work has implicated two further classes of non-coding RNA—circular RNAs (circRNAs) and small nucleolar RNAs (snoRNAs)—as regulators of the mammary response to infection. Although these molecules remain comparatively understudied in dairy cattle, they appear to participate in the same signaling networks as the better-characterized non-coding species, thereby broadening the catalog of candidate biomarkers and therapeutic targets. Parallel discoveries in the m6A epitranscriptome further indicate that the small set of m6A-related findings introduced in Section 3.2 is part of a much broader regulatory landscape spanning multiple chemokines, pathogens, and reader/eraser enzymes.

Circular RNAs as pathogen-specific regulatory species: Two parallel investigations have profiled the bovine circRNA landscape during pathogen challenge. In an *Escherichia coli*-injection model, RNA-seq of infected versus healthy mammary tissue identified 164 differentially expressed circRNAs (92 downregulated, 72 upregulated), with Gene Ontology enrichment in Ras protein signal transduction, cytoplasmic vesicle components, and enzyme binding, and KEGG enrichment in the phagosome signaling pathway; seven mastitis-associated circRNAs (novel_circRNA_0000128, _0011103, _0012656, _0015099, _005648, _000074, and _0011796) were validated by qRT-PCR [125]. In a complementary *Staphylococcus aureus* challenge model (10^5^ CFU/mL versus PBS), 202 differentially expressed circRNAs were recovered (105 up- and 97 downregulated), with enrichment in transcription by RNA polymerase II, transcription-factor complexes, oxidoreductase activity, thyroid-hormone signaling, FoxO signaling, and the cell cycle, and 7 candidate biomarkers (novel_circ_0016953, _0001266, _0015099, _0008169, _0001807, _0016220, and _0009731) nominated for further mechanistic dissection [126]. The non-overlapping circRNA signatures between *E. coli*- and *S. aureus*-challenged tissues mirror the pathogen-specificity already observed at the miRNA and lncRNA levels, and reinforce the case that pathogen-discriminating ncRNA panels could supplement clinical and bacteriological diagnosis.

Small nucleolar RNAs as a previously overlooked layer: Direct evidence linking snoRNAs to bovine subclinical mastitis has been provided by a small-RNA sequencing study of milk somatic cells from cows positive for *S. aureus* or *Staphylococcus chromogenes*, in which differential expression analysis identified 21 and 20 mastitis-associated snoRNAs, respectively [127]. Several differentially expressed species—including SNORA79 and SNORA1—are predicted to guide pseudouridylation and 2′-O-methylation at sites on 18S and 28S rRNA, implying that the inflamed mammary gland modulates ribosome composition and translational specificity during infection. Network analysis revealed that snoRNA-correlated mRNAs—among them *CXCL8*, *IL6R*, *STAT3*, *NFKB2*, *MYD88*, and *CD40*—are significantly enriched in NF-κB, JAK-STAT, and TNF signaling, and seven hub snoRNAs per pathogen group (for example, SNORA66, SNORD107 and SNORA79) each correlate with more than thirty immune-related transcripts. These findings position snoRNAs as plausible regulatory contributors to host–pathogen interactions and add a translation-level dimension to the ncRNA landscape of mastitis.

**Figure 2:**
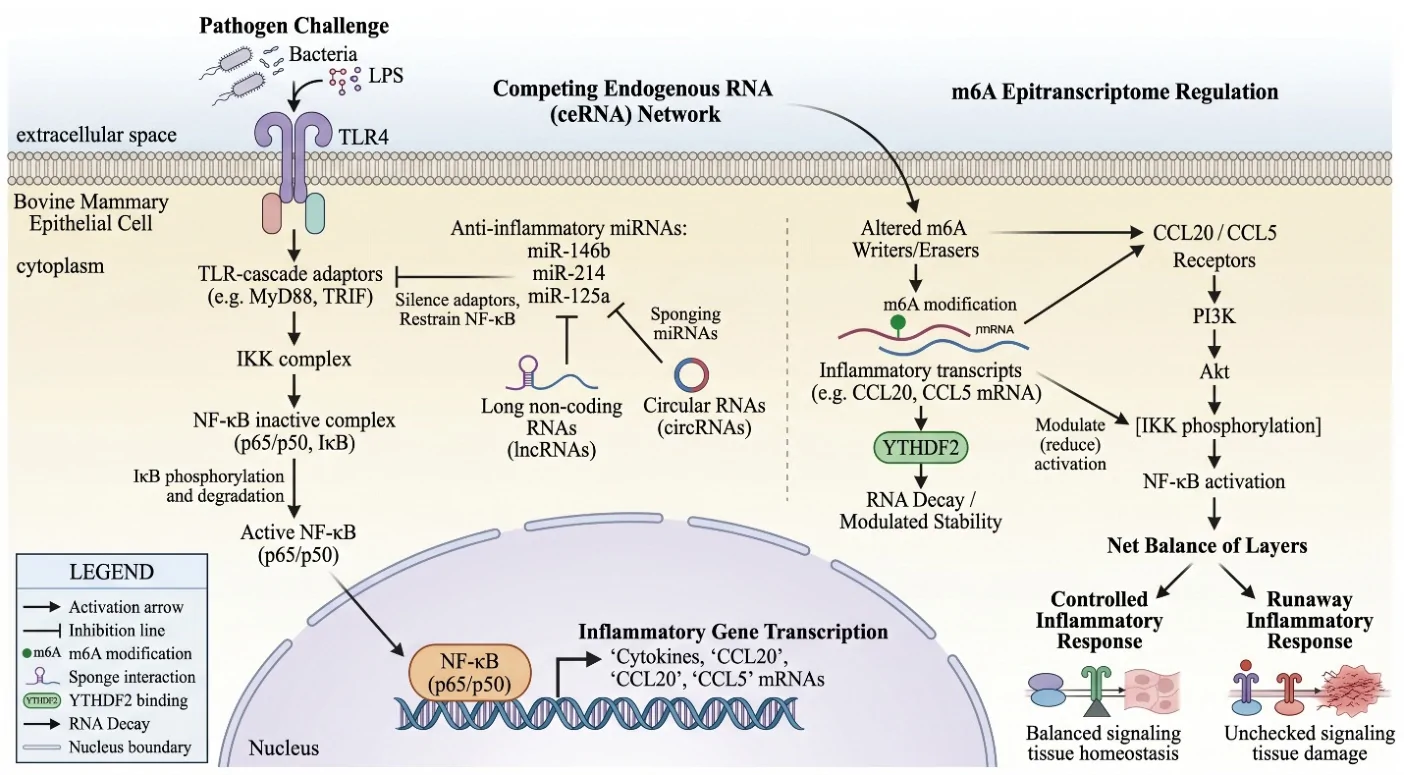
Convergence of non-coding RNAs and the m6A epitranscriptome on the TLR4/NF-κB inflammatory axis in bovine mammary epithelial cells. Anti-inflammatory microRNAs (e.g., miR-146b, miR-214, miR-125a) restrain NF-κB signaling by silencing TLR-cascade adaptors, while long non-coding RNAs and circular RNAs act as competing endogenous RNAs that sponge these microRNAs and fine-tune the response. In parallel, pathogen challenge alters the m6A modification of inflammatory transcripts, such as *CCL20* and *CCL5*, in a YTHDF2-dependent manner, thereby modulating their stability and downstream PI3K/Akt/NF-κB activation. The net balance of these layers determines whether the mammary gland mounts a controlled or a runaway inflammatory response.

An expanded m6A epitranscriptome converging on chemokine regulation: The m6A observations introduced in Section 3.2 are part of a broader, rapidly accelerating literature. Direct RNA sequencing (DRS) of *S. aureus*-challenged bMECs has identified 178 differentially expressed isoforms enriched in TNF, IL-17, and NF-κB signaling, of which 56.18% carried m6A modifications and showed highly significant expression changes between infected and control cells [128]. Among the 18 m6A-influenced inflammatory candidates, the chemokine *CCL20* emerged as the most prominent: *S. aureus* infection increased m6A modification of *CCL20* mRNA and enhanced its stability through an m6A–YTHDF2-dependent mechanism, with *YTHDF2* itself downregulated in infected cells. Silencing *YTHDF2* further elevated *CCL20* expression alongside *IL-6* and *TNF-α* and reduced anti-inflammatory IL-10 and was accompanied by increased protein expression and phosphorylation of PI3K, Akt, and NF-κB, establishing a coherent m6A–YTHDF2–CCL20–PI3K/Akt/NF-κB axis as a driver of *S. aureus* mastitis susceptibility [128] (Figure 2). Notably, *GADD45A* and *GADD45B* were also recovered as differentially expressed genes in this dataset, dovetailing with the identification of *GADD45A* as an epigenetically regulated DNA-demethylation factor in the methylome work of Dwivedi and colleagues [42], and pointing to cross-talk between DNA methylation and RNA modification systems during mastitis.

Pharmacological modulation of the same m6A machinery may offer a route to mastitis therapy. In quercetin-treated *S. aureus*-induced bMECs and a murine model, DRS revealed that multiple genes in the TNF and IL-17 pathways central to quercetin’s anti-inflammatory action were under m6A control, and quercetin specifically modulated CCL5 expression—an important chemokine in inflammatory recruitment—through m6A methylation mediated by *YTHDF2* [129]. RNA-seq further showed that quercetin altered genes involved in inflammation, extracellular matrix regulation, and matrix metalloproteinase activity (including *MMP3*, *MMP1*, *MMP1A*, and *IGFBP3*), and disrupted *S. aureus* adhesion to bMECs and biofilm formation. The convergence of two independent BMEC studies on *YTHDF2*-dependent chemokine regulation [128,129] consolidates *YTHDF2* as a tractable epitranscriptomic target for both diagnosis and intervention.

A systems-level perspective on the broader machinery is provided by an integrative analysis of 80 RNA-modification-related genes (RMRGs)—including writers, readers, and erasers of m6A and related modifications—in bovine *S. aureus* mastitis, combining public RNA-seq datasets with newly generated data [26]. RMRG expression profiles cleanly discriminated infected from control samples by principal-component analysis; weighted gene co-expression network analysis identified modules of co-regulated RMRGs, and integration with bovine QTL and transcriptome-wide association data linked specific RMRGs to immune-related complex traits. Functional interference experiments targeting the m6A demethylase *FTO*, combined with publicly available MeRIP-seq data from MAC-T cells, showed that FTO significantly modulates the expression of both m6A-related and other RNA-modification-related genes, including *NSUN2*, *CPSF2*, and members of the *METTL* family, indicating extensive crosstalk between m6A and other epitranscriptomic systems during mastitis. Collectively, the circRNA, snoRNA, and broader m6A work surveyed here reinforces the view that a comprehensive epigenetic biomarker panel for bovine mastitis will need to integrate multiple non-coding RNA classes alongside DNA methylation, miRNA, lncRNA, and histone-modification readouts. A clear hierarchy of evidence should temper this integration. Among the non-coding RNA classes, only a small number of relationships have been closed experimentally, notably the CMR/miR-877/FOXM1 axis, the DNMT1/miR-16b/YAP1 axis, and the *YTHDF2*-dependent control of *IER3* and *CCL20*, whereas the great majority of circRNA, snoRNA, and m6A findings rest on differential-expression profiling, network reconstruction, or computational target prediction rather than functional perturbation. The circRNA and snoRNA literatures in particular comprise almost entirely single descriptive studies per pathogen, without independent replication, so these species are best regarded at present as promising leads rather than validated biomarkers.

## 4. Histone Modifications, Limitations, and Future Perspectives

### 4.1. Histone Acetylation and HDAC Inhibition

Histone post-translational modifications are reversible chemical marks—principally lysine acetylation and lysine methylation—on the N-terminal tails of histone proteins that alter chromatin accessibility and recruit reader proteins to activate or repress transcription. In bMECs, exposure to LPS reduces global histone H3 acetylation, which in turn downregulates lactation-related genes such as *ACACA*, *FASN*, and *S6K1*. Pre-treatment with the histone deacetylase (HDAC) inhibitor sodium butyrate restores H3 acetylation and rescues gene expression, illustrating how environmental endotoxin exposure and dietary interventions converge on the same epigenetic machinery [130].

Direct therapeutic potential for HDAC inhibition has also been demonstrated at the level of innate antimicrobial defense: pharmacological inhibition of histone deacetylase activity increases the expression of β-defensin in bovine mammary cells and is predicted to bolster host resistance to intramammary infection [27]. Together, these observations support a dual rationale for HDAC-modulating interventions—restoring lactational gene expression silenced during endotoxin challenge while simultaneously reinforcing antimicrobial peptide output—and provide proof of principle that chromatin-targeting compounds can be repurposed for mastitis management.

### 4.2. Histone Demethylases as Therapeutic Targets

Histone demethylases have recently emerged as particularly attractive epigenetic targets in bovine mastitis, because both jumonji-domain and amine-oxidase-type enzymes can be inhibited by small molecules with established pharmacology. The H3K27me3-specific demethylase JMJD3 (KDM6B) is markedly upregulated in injured mammary glands during LPS-induced mastitis in mouse models, and pharmacological inhibition of JMJD3 with GSK-J1 significantly alleviates disease severity in both in vivo and in vitro systems [131]. Mechanistically, JMJD3 inhibition allows the repressive H3K27me3 mark to accumulate at the promoters of inflammatory genes, directly suppressing their transcription, and additionally decreases TLR4 expression and downstream NF-κB proinflammatory signaling, with corresponding reductions in *Tnfa*, *Il1b*, and *Il6* induction. Importantly, this mechanism dovetails with the in vivo observation that the repressive H3K27me3 mark is upregulated in peripheral blood lymphocytes from cows with naturally acquired *S. aureus* mastitis and correlates with downregulation of inflammatory-response genes [132], suggesting that JMJD3-targeted demethylation reactivation may be a relevant pathogenic axis in cattle as well as in murine models.

A parallel line of evidence implicates the H3K4/H3K9 demethylase LSD1 (KDM1A). LSD1, the first identified histone demethylase, is significantly upregulated in response to LPS-induced inflammation in mouse mammary epithelial cells, and its pharmacological inhibition with GSK-LSD1 elevates H3K4me2 and H3K9me2 levels, confirming the enzyme’s on-target activity in this context [133]. LSD1 inhibition reduces inflammatory cell recruitment, attenuates mammary tissue damage, and suppresses NF-κB signaling, with corresponding decreases in TNF-α, IL-6, and IL-1β production. Together, the JMJD3 and LSD1 findings establish histone demethylases as a tractable second-generation epigenetic-target class for mastitis intervention, alongside HDAC modulators, and define a rationale for testing structurally related inhibitors (and indeed methyl-donor or methyltransferase-modulating nutraceuticals) in dairy production settings.

Two cautions qualify this therapeutic optimism. First, much of the histone-modification evidence in mastitis derives from murine models and a small number of in vitro bovine experiments rather than from dairy cattle in situ, and the histone layer is consequently the least developed of the four reviewed here. The bovine-specific data, confined largely to LPS-induced H3 deacetylation, sodium-butyrate-mediated rescue of lactation genes, β-defensin induction after HDAC inhibition, and the H3K27me3 signal in peripheral blood lymphocytes of S. aureus-infected cows, remain too sparse to support firm mechanistic generalization, and direct profiling of histone acetylation and methylation in bovine mammary tissue across defined pathogens is a clear priority. Second, the practical translation of HDAC and histone-demethylase inhibitors to dairy production faces obstacles the preclinical literature does not address: these compounds act broadly across the genome and carry a risk of off-target effects, their safety in lactating animals and the potential carry-over of residues into milk are unstudied, no such agent is currently licensed for food-producing species, and the cost of repeated pharmacological intervention is difficult to justify against inexpensive management alternatives. Chromatin-targeting therapy for mastitis therefore remains a proof of concept rather than a near-term tool.

### 4.3. Hormonal Modulation of Histone Marks During Infection

Reproductive hormones constitute an additional regulatory layer shaping the epigenetic competence of the mammary gland during infection. In bMECs, the principal hormonal target tissue, combined physiological prolactin (bPRL, 5 ng/mL) and 17β-estradiol (E2, 50 pg/mL) reduce *S. aureus* internalization by ~50%—an effect linked to an ~80% decrease in integrin α5β1 and an ~25% increase in TLR2 membrane abundance—while enriching the activating H3K9ac mark at 12 h (concurrent with reduced HDAC activity) and the repressive H3K9me2 mark (in association with downregulated KDM4A) [134]. In contrast, bPRL acting alone produces a persistence-favoring profile, decreasing H3K9ac (~20%) and increasing H3K9me2 (~50%) alongside elevated HDAC activity, suppressing *S. aureus*-driven H3K9ac and H3K9me2 enrichment at the *IL-1β* and *IL-10* promoters and downregulating infection-responsive miRNAs (Let-7a-5p, miR-21a, miR-30b, miR-155, miR-7863), collectively favoring immune evasion [135]. A comparable hormone-driven reprogramming operates in primary bovine macrophages, where bPRL and E2 differentially modulate cytokine, chemokine, antimicrobial peptide, and miRNA expression, promote global H3 and H3K9 acetylation, alter HDAC activity, and reshape chemotaxis and phagocytosis consistent with these observations. The repressive H3K27me3 mark is elevated in peripheral blood lymphocytes from cows with naturally acquired *S. aureus* mastitis [136], and correlates with downregulation of immunoregulatory genes, including *IL-10* and *PTX3* [132]. Together, these findings establish that the lactational hormonal milieu directly shapes the histone-modification landscape of mammary epithelial and immune cells, providing a mechanistic basis for the parity- and lactation-stage-dependent variation in mastitis susceptibility.

### 4.4. Current Limitations

Despite rapid progress, several limitations constrain the translation of epigenetic markers into routine practice. Most studies remain limited by small sample sizes, breed-specific designs (predominantly Holstein and a few European breeds), and a focus on a narrow range of pathogens. The choice of biological sample—peripheral blood, milk somatic cells, exosomes, or biopsied mammary tissue—strongly influences which marks are detected and how reproducibly. Cause-and-effect relationships between specific marks and resistance phenotypes are rarely established with full functional validation, and the heritability and inter-generational stability of mastitis-associated marks have not been characterized across diverse production systems. Finally, standardization of laboratory protocols, bioinformatics pipelines, and reference methylomes remains incomplete. Several further obstacles deserve explicit mention. Causal inference is the central difficulty, because the overwhelming majority of methylome–transcriptome and miRNA–mRNA studies report correlation and only a handful have shown that manipulating a mark alters the phenotype. Bulk-tissue and bulk-cell assays conflate genuine per-cell epigenetic change with shifts in cell-type proportion, a particular problem in milk somatic cells and blood, where the inflammatory infiltrate itself varies with disease state, so single-cell and cell-sorted approaches will be needed to attribute marks to defined populations. Batch effects across sequencing runs, library kits, and laboratories can rival the biological signal in omics datasets, yet are seldom formally modeled, and the field still lacks agreed reference epigenomes and benchmark datasets against which new bovine studies could be calibrated.

A further conceptual gap is that this literature has been written almost entirely from the host’s perspective, treating epigenetic change as a defensive response while largely overlooking the possibility that mastitis pathogens actively reprogram the host epigenome to favor their own survival. The principle is well established in other host–pathogen systems, in which bacterial effectors and metabolites directly engage the host chromatin machinery, and fragments of the same logic are already visible in bovine mastitis. Staphylococcus aureus upregulates DNMT1, which hypermethylates and silences the protective miR-16b locus, and the same pathogen downregulates the m6A reader YTHDF2 to destabilize IER3 while stabilizing CCL20, in each case shifting the epithelial response in a direction the bacterium appears to exploit. Whether specific virulence factors, such as secreted toxins or surface proteins, bind or modulate host DNMTs, HDACs, or histone demethylases in cattle remains essentially untested. Distinguishing pathogen-imposed reprogramming from host-initiated defense is more than a semantic matter, because it determines whether a given mark should be reinforced or blocked therapeutically, and it should be a priority for mechanistic work, ideally using defined bacterial mutants to separate the two. A comparative overview of evidence maturity, preferred sampling matrices, key strengths, and limitations across all seven biomarker classes reviewed in this article is provided in Table 3.

### 4.5. Future Perspectives

Several directions are likely to advance the field. First, multi-omic integration of methylomes, transcriptomes, microRNAomes, and epitranscriptomes (m6A) across well-phenotyped cohorts will help disentangle causal from correlative marks. Second, the development of low-cost, targeted methylation and milk-miRNA assays could deliver chair-side or in-line diagnostics for early detection of subclinical mastitis. Third, the moderate heritability of methylation marks invites their inclusion in genomic prediction models—an approach sometimes called epigenomic selection—particularly for low-heritability disease traits where conventional selection has plateaued. Fourth, the reversibility of epigenetic marks opens the door to nutritional and pharmacological interventions (such as methyl-donor supplementation, HDAC inhibitors, histone-demethylase inhibitors, m6A modulators, or miRNA mimics and antagomirs) aimed at training the udder for greater resilience. Fifth, targeted epigenome editing—exemplified by the pdCas9-C-Tet1-SgRNA 2.0 demethylation of the *AKT1* promoter in *S. aureus*-challenged BMECs—offers a route from descriptive biomarker discovery to mechanistic intervention. Finally, broader coverage of breeds and pathogens, combined with longitudinal sampling, will be essential to translate proof-of-concept biomarkers into validated tools for the global dairy industry.

A recurring theme across Section 2 and Section 3 is the gap between correlation and causation. Large integrative datasets, such as the 62,940 differentially methylated cytosines reported in Vrindavani cattle and the many paired methylome–transcriptome and miRNA–mRNA catalogues, establish association but not mechanism, and only a small number of studies have closed the loop with experimental perturbation; namely, the targeted demethylation of the AKT1 promoter, the validation of the CMR/miR-877/FOXM1 sponge axis, and the functional dissection of YTHDF2-dependent transcript stability. Bridging this gap is the single most important methodological priority for the field. Practical routes include CRISPR-based epigenome editing to write or erase a candidate mark at its endogenous locus, miRNA mimics and inhibitors to test sufficiency and necessity, allele-specific and longitudinal designs that exploit natural variation, and Mendelian-randomisation-style analyses that use genetic instruments to infer the direction of causality. Until more marks survive this kind of test, most reported epigenetic signatures should be treated as candidate biomarkers of immune state rather than as established drivers of resistance or susceptibility.

The prospect of epigenomic selection, although attractive, raises implementation questions that remain largely unanswered. Incorporating methylation or other epigenetic marks into genomic prediction would require assays cheap and robust enough to run at population scale, analytical pipelines able to integrate epigenetic with conventional SNP information without inflating dimensionality, and, most fundamentally, evidence that the selected marks are stable enough across parities and generations to deliver durable genetic gain. Because many mastitis-associated marks are environmentally responsive and reset within a lifetime, their value may lie more in within-animal monitoring and management decisions than in heritable selection, and the balance between these two uses needs empirical resolution. This also argues for situating epigenetic markers within the wider system of udder-health management, in which milking physiology, teat-tissue biomechanics, teat-canal integrity, and automated milking systems shape the environmental exposures that the mammary epigenome records, so that biological, management, and technological factors are integrated rather than studied in isolation [28].

## 5. Conclusions

Epigenetic markers add a complementary dimension to the genetic improvement of mastitis resistance, capturing the cumulative imprint of environmental and pathogen exposures on the regulatory architecture of immune and mammary genes. DNA-methylation panels and discriminant methylation haplotype blocks, milk-borne and exosomal miRNA signatures, lncRNA-, circRNA-, and snoRNA-based readouts, m6A profiles, and histone-mark dynamics together offer a richer and more responsive readout of udder health than DNA sequence alone. Continued methodological refinement and large-scale validation will determine how rapidly these markers move from discovery to deployment in dairy herds. It should be emphasized, however, that with few exceptions, these markers remain at the discovery stage. Large-scale validation in independent cohorts, demonstrated reproducibility across breeds and production systems, and evidence of predictive value under field conditions are all necessary before routine implementation in dairy herds becomes realistic, and the most useful near-term contribution of this work is likely to be improved mechanistic understanding and non-invasive monitoring rather than immediate selection or therapy.

## Figures and Tables

**Table 3:** Evidence maturity, strengths, and limitations of epigenetic and epitranscriptomic biomarker classes in bovine mastitis.

Marker Class	Evidence Maturity	Best-Validated Candidates	Preferred Matrix	Key Strengths	Key Limitations
DNA methylation	Moderate to high	JAK2, STAT5A and CD4 promoters; ENOPH1; dMHBs (CD48, IL10); FHIT; AKT1 (functionally demethylated)	Blood, milk somatic cells, mammary tissue	Quantitative and relatively stable; anchored to GWAS and QTL; one functional proof of concept (AKT1)	Small cohorts; breed- and matrix-dependent discordance; predominantly correlative; platform heterogeneity
microRNAs	Moderate to high	miR-223, miR-146a/b, let-7, miR-125a, miR-16b, miR-877 (several functionally validated)	Milk, milk exosomes, serum, blood	Non-invasive; nuclease-resistant; reproducible core regulators; several causal axes established	Modest individual ROC (about 0.70 to 0.74); inconsistent normalization; small validation cohorts
lncRNAs	Moderate	BMNCR, CMR, lncRNA-TUB, LRRC75A-AS1, H19 (functionally tested); PRANCR, TNK2-AS1 (QTL-anchored)	Mammary tissue, bMECs, blood	Mechanistically deep ceRNA hubs; several co-localize with mastitis QTL	Most loci only differentially expressed; context-dependent or dual roles; little field validation
circRNAs	Low (exploratory)	Pathogen-specific panels distinguishing *E. coli* from *S. aureus*	Mammary tissue	Pathogen-discriminating signatures complementary to bacteriology	Single descriptive study per pathogen; no functional validation or independent replication
snoRNAs	Low (exploratory)	SNORA79, SNORA1, SNORD107 (correlative hubs)	Milk somatic cells	Add a translation-level regulatory dimension via rRNA modification	One study; predicted rather than proven targets; no validation
m6A epitranscriptome	Low to moderate	YTHDF2/IER3 and YTHDF2/CCL20 (functionally validated); FTO crosstalk	bMECs, mammary tissue	Defined reader and eraser axes; a pharmacological handle (quercetin)	Few cohorts; largely in vitro; an emerging and still-narrow field
Histone modifications	Low	H3 acetylation and sodium butyrate; JMJD3/GSK-J1 and LSD1 (mostly murine); H3K27me3 in cattle blood	Mammary tissue, blood lymphocytes, bMECs	Pharmacologically tractable enzyme targets	Heavily reliant on murine models; sparse bovine data; safety, regulatory, and economic barriers

## Data Availability

No new data were created or analyzed in this study. Data sharing is not applicable to this article.

## Abbreviations

bMECs, bovine mammary epithelial cells; ceRNA, competing endogenous RNA; circRNA, circular RNA; CMT, California Mastitis Test; CoNS/CoPS, coagulase-negative/positive staphylococci; DMC, differentially methylated cytosine; DMEG, differentially methylated and expressed gene; DMG, differentially methylated gene; DMR, differentially methylated region; dMHB, differentially methylated haplotype block; DNMT, DNA methyltransferase; EM-seq, enzymatic methyl sequencing; EV, extracellular vesicle; GWAS, genome-wide association study; HDAC, histone deacetylase; lncRNA, long non-coding RNA; LPS, lipopolysaccharide; LTA, lipoteichoic acid; m6A, N6-methyladenosine; MeDIP-seq, methylated DNA immunoprecipitation sequencing; miRNA, microRNA; ncRNA, non-coding RNA; NF-κB, nuclear factor kappa B; piRNA, PIWI-interacting RNA; QTL, quantitative trait locus; ROC, receiver operating characteristic; RRBS, reduced-representation bisulfite sequencing; SCC, somatic cell count; SCM, subclinical mastitis; SCS, somatic cell score; snoRNA, small nucleolar RNA; TE, transposable element; TLR, Toll-like receptor; WGMS, whole-genome methylome sequencing.
